# Side Effects Associated With Respiratory Syncytial Virus Prefusion F (RSVpreF) Maternal Vaccination: A Scoping Review

**DOI:** 10.7759/cureus.88162

**Published:** 2025-07-17

**Authors:** Mabel Palmero, Roberto A Martin, Benjamin Olson, Lindsey Millares, Macey Melinek, Delaney Waggoner, Caitlin Higginbotham, Alia E Gali, Sophia M Kershner, Joseph S De Gaetano

**Affiliations:** 1 Family Medicine, Nova Southeastern University Dr. Kiran C. Patel College of Osteopathic Medicine, Fort Lauderdale, USA

**Keywords:** adverse effects, female, infant, pregnancy, respiratory syncytial virus vaccination, rsvpref, treatment outcome

## Abstract

Respiratory syncytial virus (RSV) is a leading cause of lower respiratory tract infections (LRTIs), with significant morbidity and mortality in infants. In August 2023, the Pfizer RSVpreF (respiratory syncytial virus prefusion F) vaccine was approved in the United States for maternal immunization, aiming to reduce RSV-related illness in neonates. The vaccine has proven efficacy in preventing severe LRTI in infants. However, limited research exists on its potential side effects, particularly on maternal health outcomes. This scoping review aimed to assess the extent and type of evidence available regarding safety and maternal side effects of the Pfizer RSVpreF vaccine when administered during pregnancy. A systematic search was conducted across six databases, including Ovid MEDLINE, Embase, CINAHL (Cumulative Index to Nursing and Allied Health Literature), Cochrane Central, Web of Science, and ClinicalTrials.gov, for peer-reviewed studies published between January 2020 and September 2024. Studies focusing on maternal outcomes following RSVpreF vaccine administration in pregnancy were included. Data extraction and analysis were performed following the Joanna Briggs Institute (JBI) methodology for scoping reviews. Of 1,259 initially identified studies, five met the inclusion criteria. The studies, spanning 2020-2024, included randomized controlled trials and a retrospective cohort study, primarily regarding high-income populations. Across the studies, findings indicate that the RSVpreF vaccine is generally well tolerated, with the most common side effects being mild-to-moderate local injection site reactions. Adverse events, including preeclampsia and gestational hypertension, were reported inconsistently across studies. A recent cohort study suggested a possible association between vaccine administration and hypertensive disorders of pregnancy (HDP), though findings remain inconclusive. Overall, the Pfizer RSVpreF vaccine appears to be well tolerated in pregnant women, but gaps in maternal safety data warrant further research. Future studies should prioritize high-risk patients and underrepresented populations, long-term maternal health outcomes, and potential associations with hypertensive disorders to ensure comprehensive vaccine safety and equitable access.

## Introduction and background

Respiratory syncytial virus (RSV) has historically been well known as a primary cause of ​lower respiratory tract infections (LRTIs). ​​​It is thought that in the United States, virtually all children will have experienced RSV by age two [1]. Though self-limiting in adults, RSV can cause severe illness in individuals who are immunocompromised, notably ​in premature infants, young children,​ and pr​egnant women [2]. In 2015, RSV-associated LRTIs in children under five accounted for approximately 3.2 million hospital admissions, with 5​9,500 resulting in death. For infants under six months, deaths occurred in 45% of all admitted cases [3]. Newborns are thought to ​have partial immunity from the ​virus from maternal RSV ​antibodies. Between two and four months of age, RSV-associated morbidity and​ mortality peaks due to a decrease in maternal antibody levels and a lack of replacement by endogenous antibodies [4,5]. In addition to its impact in infancy, RSV has also been implicated as a common source of acute respiratory infection during pregnancy [6]. RSV infection during pregnancy may also be linked to a greater incidence of hospitalizations and complications, such as preterm delivery [7-9]. As the full implications of RSV infection in pregnancy ​are unknown due to a current gap in maternal RSV ​research, it is suggested that the prevalence and complications of RSV in pregnancy are underreported [10]. The impact of RSV is not limited to the acute setting. Some studies have also indicated that it may be associated with other long-term health outcomes. Researchers have found that RSV bronchiolitis in infancy could play a potential role in the development of childhood asthma [11,12]. Due to RSV’s high incidence rate and its many short-term and long-term complications, the development of the RSV vaccine was crucial [13]. The creation of a maternal RSV vaccine may not only be beneficial in its ability to provide protection to infants but may also offer protection to pregnant women.

​​The earliest RSV vaccine to undergo ​clinical investigation was a formalin-inactivated vaccine​ developed in the 1960s for infants. This vaccine (formalin-inactivated RSV) proved to be both non-efficacious and unsafe. This stalled the research surrounding the vaccine for decades [4,14]. In 1998, a monoclonal antibody (palivizumab) was approved for infants with very specific risk factors for severe disease. There proved to be serious limitations and moderate efficacy with this treatment [5]. Development of other various types of RSV vaccines occurred: ​​​particle-based, vector-based, live-attenuated, subunit vaccines, and mRNA vaccines [​4]. Eventually, subunit vaccines ​​​began showing superior efficacy, and three main manufacturers, Pfizer, GSK, and Novavax, began working on developing an RSVpreF (respiratory syncytial virus prefusion F) formulation for use during pregnancy [15]. On August 21st, 2023, the Pfizer ​RSVpreF vaccine received approval in the USA for​ use in maternal immunization. The Pfizer RSVpreF vaccine’s administration during 32 to 36 weeks of gestational age was approved as a prophylaxis against the development of LRTIs ​in infants from birth up until six months [5].

RSVpreF vaccines contain the RSV F glycoprotein stabilized in its prefusion (pre-F) state. By focusing on the ​pre-F glycoprotein, these vaccines are able to engage cell-mediated as well as humoral immunity, promoting memory B and T cell formation, and leading to the creation of maternal antibodies [4]. Current research on this subunit vaccine has focused on its ability to prevent RSV infection in infancy and its efficacy in transferring maternal antibodies to the neonate. This formulation has been shown to be effective in preventing severe LRTI in infants, increasing levels of RSV-specific antibodies in maternal recipients of the vaccine, and successfully transferring transplacental RSV-neutralizing antibodies to infants [16,17]. Initial studies have concluded that the administration of RSVpreF vaccines to pregnant women is well tolerated with no apparent safety concerns other than increased short-term postvaccination reactogenicity [16,17]. However, the current data are unclear on the relationship between vaccine administration and the risk of preterm birth. Some researchers have concluded that the vaccine does not increase risk for preterm birth after adjusting for confounders [16]. Others have found an increased risk [5,18,19]. Despite their near identical formulation, only GSK vaccine trials have been stopped due to a safety signal of increased preterm births and neonatal deaths, while the Pfizer vaccine has been approved for use [19,20].

The Centers for Disease Control and Prevention reported in the 2023-2024 United States RSV season that only 33% of pregnant women eligible to receive the Pfizer RSVpreF vaccine chose to be vaccinated [21]. Maternal confidence in vaccine safety has long been identified as a major factor influencing vaccine hesitancy and may play a role in the immunization patterns seen during this vaccine’s first available season post approval [22]. A review of current literature shows that previously conducted systematic reviews have focused on maternal vaccination with bivalent RSVpreF formulations from Pfizer and GSK and emphasized the vaccine’s efficacy in preventing LRTIs in the neonatal period [23]. This review differs in that it will focus exclusively on maternal immunization with the Pfizer RSVpreF formulation, as it is the only formulation that has received FDA approval. Additionally, this scoping review will focus on the maternal side effects of vaccination alone. A scoping review establishing the maternal safety profile of the Pfizer RSVpreF vaccine is essential to determine the need for further research and help address vaccine hesitancy in eligible recipients.

Although ​the efficacy of the​ RSVpreF vaccine in lowering RSV infection in neonates has been established, research on the potential side effects associated with the administration of the vaccine has been limited. The most reported event has been injection site reactions [16]. Given the relative newness of the vaccine, little is known about the effects of the vaccine on maternal and perinatal health outcomes. A current review of the existing literature demonstrates​ that there is a significant gap in our understanding of the potential side effects of the newly approved Pfizer maternal RSV vaccine. A scoping review will be conducted to identify the current breadth of literature regarding the maternal safety of Pfizer RSVpreF vaccination and to identify specific side effects that will merit further study.

## Review

Methods

The protocol for this scoping review was created in accordance with the methodology outlined by The Joanna Briggs Institute (JBI) Scoping Review Methodology and the Preferred Reporting Items for Systematic Reviews and Meta-Analyses extension for Scoping Reviews (PRISMA-ScR) guidelines [24,25].

Eligibility Criteria

Inclusion for this review encompassed studies focused on maternal participants who received the new Pfizer RSVpreF vaccine (Pfizer, New York, NY) as part of routine prenatal care. Only articles published between January 2020 and September 2024 were included, as initial RSVpreF vaccine human trials in pregnant women began in June 2020. Studies not including administration of the vaccine in pregnant women, vaccines not produced by Pfizer, and studies that ignored maternal outcomes in favor of neonatal outcomes were excluded. This scoping review considered randomized controlled trials, non-randomized controlled trials, cohort studies, and case-control studies. Systematic reviews and meta-analyses, as well as studies not yet completed, were excluded. The level of evidence was not considered as an exclusion criterion due to the novelty of the topic and the limited research available at the time.

Search Strategy and Information Sources

In conjunction with a medical research librarian, the first (MP) and second (RM) authors of this review created a search strategy that included terms related to the RSV vaccine extracted from MeSH and relevant articles, titles, keywords, and abstracts identified via Embase. The identified terms included were “Respiratory Syncytial Virus Vaccines” (a MeSH term) and “Pregnancy” (a MeSH term) as well as additional key terms related to the topic. This search strategy was used to conduct a systematic search across six peer-reviewed databases that included Ovid MEDLINE, Embase, CINAHL (Cumulative Index to Nursing and Allied Health Literature), Cochrane Central, Web of Science, and ClinicalTrials.gov. The aim of the diversity of databases was to ensure all relevant articles on the topic were identified and to ensure this scoping review was representative of the current state of research on the topic. A search was conducted on September 14th, 2024, and was limited to peer-reviewed studies published in the English language, whose full text and abstracts were available in full via the Nova Southeastern University (NSU) database. The full search strategies can be found in the Appendix.

Selection of Sources of Evidence and Data Charting

An initial screening of titles was conducted by authors four through six to identify potentially relevant papers. After duplicate removal using EndNote (Clarivate, Philadelphia, PA), a blinded tier one review of titles and abstracts of potentially relevant articles was conducted by two independent reviewers (MP and RM) using Rayyan software (Rayyan, Cambridge, MA). Titles and abstracts not meeting previously established inclusion criteria were excluded. Discordance between the blinded reviewers was resolved by a third author (BO). Full articles were then retrieved, and a blinded tier two review was conducted in Rayyan in the same fashion. The primary author developed a charting form in Microsoft Excel (Microsoft Corporation, Redmond, WA) to guide the extraction of variables (MP). Data were extracted from each article independently by authors five through nine, and discordances in included data were resolved by the first author. Data extracted from each article included characteristics such as the author, year of publication, and country of origin where the study took place. Additionally, features of each study were extracted to include details about each study’s purpose, population, and sample size, methodology, type of study design, key findings relevant to the review question, and limitations of each study.

Critical Appraisal

Although not required for scoping reviews, in an effort to ensure quality of evidence, a critical appraisal of all eligible articles identified by the tier two review was performed using the critical appraisal tools identified by the JBI checklist [26,27]. Articles were assessed in depth for bias and rigor using the appropriate JBI checklist based on study methodology. Five authors were each assigned one article to assess in depth for bias and rigor using the appropriate JBI checklist based on study methodology (authors five through nine). The studies were then categorized into three risk levels: scores below 50% indicating high risk, scores between 50% and 70% indicating moderate risk, and scores above 70% indicating low risk. All articles that scored above 70% in the JBI criteria were included in the review. The relevance and quality of each study were thoroughly discussed as a team to select the final articles to be included in the review, and all five identified studies met approval for inclusion [28-32]. Findings from the critical appraisal checklists can be found in Tables 1, 2.

**Table 1:** The Joanna Briggs Institute (JBI) critical appraisal checklist for randomized control trials (RCTs). Created in accordance with the JBI critical appraisal tool for the assessment of risk of bias for randomized controlled trials [27].

Criteria	Madhi et al. [28]	Justification	Simoes et al. [29]	Justification	Kampmann et al. [30]	Justification	Otsuki et al. [32]	Justification
Was true randomization used for the assignment of participants to treatment groups?	Yes		Yes		Yes		Yes	
Was allocation to treatment groups concealed?	Yes		Yes		Yes		Yes	
Were the treatment groups similar at the baseline?	Yes		Yes		Yes		Yes	
Were participants blind to treatment assignment?	Yes		Yes		Yes		Yes	
Were those delivering treatment blind to treatment assignment?	Yes		No	The study dispenser/administrator was unblinded. All other personnel (maternal participant, study coordinator, investigator, and site staff) were blinded. Contact between unblinded administrators and maternal participants was kept to a minimum.	Yes		Yes	
Were outcomes assessors blind to treatment assignment?	Yes		Yes		Yes		Yes	
Were treatment groups treated identically, other than the intervention of interest?	Yes		Yes		Yes		Yes	
Was follow-up complete, and if not, were differences between groups in terms of their follow-up adequately described and analyzed?	Yes		Yes		Yes		Yes	
Were participants analyzed in the groups to which they were randomized?	Yes		Yes		Yes		Yes	
Were outcomes measured in the same way for treatment groups?	Yes		Yes		Yes		Yes	
Were outcomes measured in a reliable way?	Yes		Yes		Yes		Yes	
Was appropriate statistical analysis used?	Yes		Yes		Yes		Yes	
Was the trial design appropriate, and did any deviations from the standard RCT design (individual randomization, parallel groups) accounted for in the conduct and analysis of the trial?	Yes		Yes		Yes		Yes	

**Table 2:** The Joanna Briggs Institute (JBI) critical appraisal checklist for cohort studies. Created in accordance with the JBI critical appraisal tool for the assessment of risk of bias for cohort studies [26].

Criteria	Son et al. [31]	Justification
Were the two groups similar and recruited from the same population?	Yes	
Were the exposures measured similarly to assign people to both exposed and unexposed groups?	Yes	
Was the exposure measured in a valid and reliable way?	Yes	
Were confounding factors identified?	Yes	
Were strategies to deal with confounding factors stated?	Yes	
Were the groups/participants free of the outcome at the start of the study (or at the moment of exposure)?	Yes	
Were the outcomes measured in a valid and reliable way?	Yes	
Was the follow-up time reported and sufficient to be long enough for outcomes to occur?	Not applicable	No follow-up was conducted as this was a retrospective observational cohort study.
Was follow-up complete, and if not, were the reasons for loss to follow-up described and explored?	Not applicable	No follow-up was conducted as this was a retrospective observational cohort study.
Were strategies to address incomplete follow-up utilized?	Not applicable	No follow-up was conducted as this was a retrospective observational cohort study.
Was appropriate statistical analysis used?	Yes	

Results

**Figure 1:**
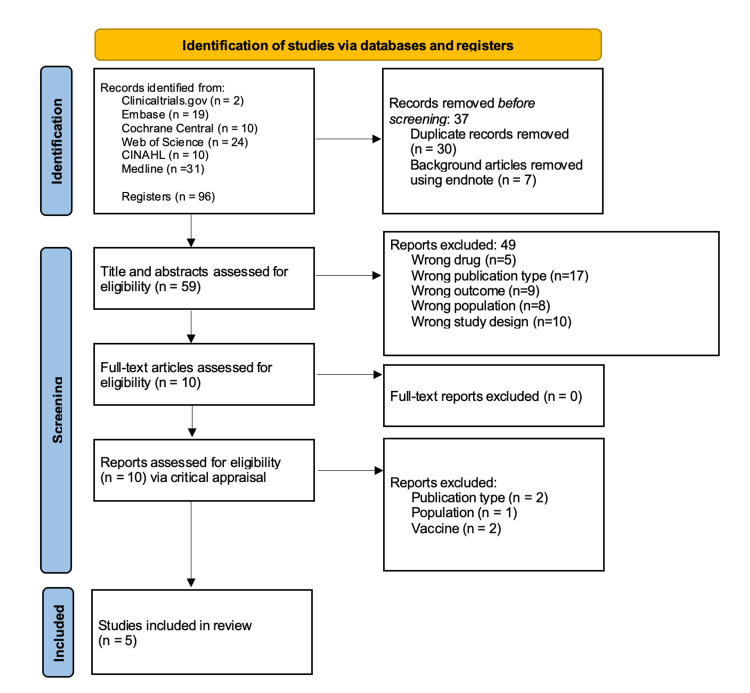
PRISMA flow diagram. PRISMA: Preferred Reporting Items for Systematic Reviews and Meta-Analyses.

After conducting searches in Ovid MEDLINE, Embase, CINAHL, Cochrane Central, Web of Science, and ClinicalTrials.gov, a total of 1,259 articles were identified whose titles and abstracts were screened to identify relevant papers. This initial review resulted in 96 articles, which were uploaded to EndNote, where 37 duplicates and background articles were removed. The remaining 59 articles were evaluated for eligibility, with 10 articles being selected for full-text review. Of these, five met the inclusion criteria and underwent critical appraisal, with consensus being reached among the first three authors [28-32]. Results of these selected articles were documented in adherence to the PRISMA-ScR guidelines and are reported in Figure 1 [25].

The studies included in this review spanned from 2020 to 2024 [28-32], with two of those being published in 2024 [31,32]. This indicates a trend of increasing research regarding the vaccine since its initial FDA approval in August of 2023. The studies differed in methodology, with one article being a randomized, observer-blind, placebo-controlled trial [28], another being a double-blind, randomized, placebo-controlled trial [30], two being analyses of randomized control trials [29,32], and the last being a retrospective observational cohort study [31].

Of five studies, two took place exclusively in the United States [29,31], one exclusively in Japan [32], and two included a range of multinational sites [28,30]. Of the two multinational studies, one included Argentina, Australia, Chile, Mexico, New Zealand, the Philippines, South Africa, Spain, Brazil, Canada, Denmark, Finland, Gambia, Japan, the Netherlands, the Republic of Korea, and Taiwan [28]. The other included Argentina, Australia, Chile, Mexico, New Zealand, the Philippines, South Africa, Spain, Bangladesh, and the United Kingdom [30]. The studies included a range of participants from 400 to over 4,000 healthy, pregnant women under 49 years old with low-risk singleton pregnancies between 28 weeks and 36 weeks of gestation. All studies included the administration of the Pfizer RSVpreF vaccine compared against a placebo control group, with one study including an additional comparison of RSVpreF vaccine with and without aluminum hydroxide [29]. The median maternal age ranged from 26 to 29 years, with the median gestational age ranging from 31.2 to 31.3 weeks [28-30,32]. A summary of the characteristics of the included studies is provided in Table 3.

**Table 3:** Summary of the included studies. RSV: respiratory syncytial virus; RSVpreF: respiratory syncytial virus prefusion F; AEs: adverse events; SAEs: severe adverse events; LRTI: lower respiratory tract infection; HDP: hypertensive disorders of pregnancy.

Citation	Country	Objective	Population	Design	Methods	Findings	Limitations
Madhi et al. (2020) [28]	Argentina, Australia, Chile, Bangladesh, Mexico, New Zealand, the Philippines, South Africa, Spain, the United Kingdom, the United States	To show the efficacy of maternal immunization with the respiratory syncytial virus (RSV) F protein vaccine for protection of infants against RSV-associated, medically significant lower respiratory tract infection up to 90 days of life.	4636 healthy women 18 to 40 years old with low-risk singleton pregnancies between 28 weeks and 36 weeks of gestation.	Randomized, observer-blind, placebo-controlled trial	The trial was conducted at 87 sites. Vaccine was administered to women with low-risk pregnancies prior to the start of seasonal RSV circulation in their location. Weekly active surveillance was conducted for 180 days after delivery to assess for lower respiratory tract infections (LRTI) symptoms. Participants were monitored for injection site reaction and systemic reactogenicity for seven days post vaccination. Evaluation for adverse events (AE) and new medical conditions was assessed throughout trial participation until six months post delivery.	The RSV vaccine was found to be well tolerated. Vaccine administration was found to be associated with local reactogenicity more than placebo administration. Systemic reactogenicity was similar in both groups. No statistically significant differences in AEs and delivery outcomes were found.	The trial is limited by its early termination due to low enrollment rates and the sponsor determining that sufficient data had been collected to assess vaccine efficacy. The study was also limited in that it was not powered enough to determine efficacy and safety by country.
Simões et al. (2022) [29]	The United States	To assess the safety of RSVpreF maternal vaccination and evaluate solicited local and systemic reactions as well as unsolicited AEs.	406 healthy women at 24 to 36 weeks of gestation, with singleton uncomplicated pregnancies and no known risk of complications.	Interim analysis of randomized controlled trial	Eligible women were randomized to receive 120 or 240 μg of RSVpreF vaccine (with or without aluminum hydroxide) or placebo. The trial assessed safety endpoints that included local reactions for seven days post vaccination via an electronic diary, and AEs and SAEs were assessed at two weeks post vaccination, four weeks post vaccination, and at the time of delivery. As this was an interim analysis of an ongoing trial, the study focused only on those participants located in the United States.	Most post vaccination reactions were mild to moderate. Participants receiving the RSVpreF vaccine containing aluminum hydroxide reported a higher incidence of local reactogenicity than those who received the RSVpreF vaccine without aluminum hydroxide. Incidence of AEs was similar between the vaccine and placebo groups.	The study was limited in that the analysis looked at only participants in the United States, and findings may not be representative of other countries. Women vaccinated between 24 and 27 weeks were underrepresented in the cohort, as many had not given birth by the data cutoff and were therefore not included in the analysis. Small sample size may limit the validity of the trial’s findings.
Kampmann et al. (2023) [30]	The United States, South Africa, Argentina, Japan, Taiwan, Spain, Gambia, Netherlands, Chile, Finland, New Zealand, Philippines, Mexico, Brazil, Denmark, Canada, Australia, Republic of Korea	To evaluate the safety and efficacy of maternal immunization with RSVpreF against LRTI in infants.	7392 healthy women, ages 49 or younger, at 24 to 36 weeks of gestation with an uncomplicated singleton pregnancy and no known risk of pregnancy complications.	Double-blind, randomized, placebo-controlled trial	The trial was conducted in 18 countries over four RSV seasons and followed infants for one to two years. Eligible participants were randomly assigned in a 1:1 ratio to receive a single intramuscular injection of 120 μg of RSVpreF vaccine (60 μg each of RSV A and RSV B antigens) or placebo. The trial assessed safety end points by monitoring reactogenicity in maternal participants for seven days post vaccination via an electronic diary. Data on maternal AEs were collected one month post injection, and SAEs were assessed six months post injection.	Local reactions were more commonly reported in the vaccine group, with the most commonly reported reaction being injection site pain. Systemic symptoms within seven days of vaccination were similar among both groups, except for headaches and muscle pain, which were more common in the RSVpreF group. No safety signals were detected in the maternal participants. The incidence of AEs one month after vaccination was similar between groups. SAEs within six months were similar between groups, with the most common being preeclampsia and fetal distress syndrome.	This trial is limited by the exclusion of women with high-risk pregnancies whose children could be at higher risk of developing LRTI associated with RSV; therefore, possibly overestimating the efficacy and safety of the vaccine. The trial included limited data from low-income countries with higher known RSV impact. The trial was insufficient in assessing vaccine efficacy by RSV subgroup.
Son et al. (2024) [31]	The United States	To evaluate the association between prenatal RSV vaccination status and perinatal outcomes among patients who delivered during the vaccination season.	2973 women who gave birth to singleton gestations at 32 weeks or later from September 22, 2023, to January 31, 2024, in two New York City hospitals.	Retrospective observational cohort study	The data were extracted from a research repository constructed from electronic health records. Patients who had given birth from September 22, 2023, to January 31, 2024, were included as they had an opportunity to be exposed to RSV during the local season. Patients with unknown gestation age or multifetal pregnancies were excluded. Data was extracted to determine which patients had received the RSVpreF vaccine during their pregnancy and to assess for preterm birth outcomes and secondary pregnancy outcomes such as hypertensive disorders of pregnancy (HDP). Statistical analyses were run to assess for correlations between vaccination and the identified outcomes.	34.5% of identified patients were found to have received the RSVpreF vaccine during pregnancy. Evidence of vaccination during pregnancy was not significantly associated with preterm birth. One statistical model found an increased risk of HDP associated with RSVpreF vaccination. Birth outcomes were similar among the groups.	Study location and patient population may not be generalizable. Due to the use of electronic health records, it is possible that records of vaccination from smaller pharmacies were not included, and some patients may have been misclassified in the analysis. The study reflected higher vaccination frequency than the national average, and its findings may not be generalizable to other cities or countries. The small sample size limits the validity of the findings.
Otsuki et al. (2024) [32]	Japan	To evaluate the safety and efficacy of maternal vaccination with the respiratory syncytial virus prefusion F vaccine in pregnant women of 24-36 weeks gestation and their infants.	462 pregnant healthy women in Japan under the age of 49 years old and between 24 and 36 weeks of gestation with uncomplicated pregnancies with no identifiable risk of future complications.	Subset analysis of a randomized, observer-blind, placebo-controlled trial	Subgroup analysis from the Maternal Immunization Study for Safety and Efficacy (MATISSE) trial focused on patients enrolled in Japan. Assessed maternal safety endpoints as local/systemic reactions seven days after vaccine administration, AEs within one month of administration, and severe adverse events (SAEs) through six months of vaccination. Participants were randomly assigned in a 1:1 distribution to receive the RSVpreF vaccine or placebo.	The RSVpreF vaccine was found to be well tolerated and safe for pregnant women and their infants participating in the trial. Local reactions were found to be mild to moderate in maternal participants. Reported adverse effects were found to be similar in the vaccinated and placebo groups within one month. No SAEs were reported within one month.	This trial was not designed for a subset analysis of the vaccine efficacy. The study was located only in Japan and may not be generalized to other countries.

For the studies included in this review, the maternal safety of RSVpreF vaccination was generally assessed via three outcomes: reactogenicity, adverse effects, and efficacy of the RSVpreF vaccine. Reactogenicity was assessed within seven days of vaccine administration and was divided into local and systemic reactions. Local reactogenicity was defined as an inflammatory response that included injection site pain, erythema, and tenderness. Systemic reactogenicity was defined as fever, fatigue, headache, nausea, muscle pain, joint pain, vomiting, and diarrhea across the five selected articles.

Across the studies, RSV vaccine administration during pregnancy was found to be generally well tolerated, with side effects being primarily mild to moderate local reactions, which were found to be more common in participants who received the vaccine compared to those in the placebo group [28-30,32]. In most studies, systemic reactogenicity was similar between vaccine and placebo groups; however, one study found that headaches and muscle pain were more common in the vaccine group [30]. When comparing vaccines formulated with aluminum hydroxide to those without, researchers found that local reactogenicity was greatest in participants who received the aluminum hydroxide formulation [29].

Discrepancies in the categorization of adverse effects were found across the included studies. Adverse effects were categorized as “adverse events” (AEs) and “serious adverse events” (SAEs) among a majority of the articles [28-31]. However, one study included an additional category of “adverse events of special interest” to highlight adverse events that may directly correlate to the RSVpreF vaccine [32]. Adverse events included pre-eclampsia, gestational hypertension, and premature delivery. One study included pre-eclampsia and premature delivery among the serious adverse events [30], rather than adverse events as seen in the remaining studies [28-30,32]. Serious adverse events were defined as postpartum hemorrhage, placental abruption, and chorioamnionitis. Generally, the incidence of AEs and SAEs was similar between vaccine and placebo groups; however, a recent study found that vaccine administration during pregnancy was associated with an increased risk of developing hypertensive disorders of pregnancy (HDP) [31]. Additionally, across all studies, birth outcomes were found to be similar between groups, and RSVpreF vaccine administration was not found to be associated with preterm birth [28-32].

There were significant gaps in the literature on the maternal side effects of RSVpreF vaccination. Among the included studies, investigative focus was placed on assessing the efficacy of vaccine administration in preventing RSV-associated LRTI in neonates. However, the studies were limited in the evaluation of the side effects of vaccine administration in maternal participants. Additionally, findings of maternal safety of vaccine administration were limited as studies excluded high-risk pregnancies and generally underrepresented low-income pregnant women [28-32]. All studies included were primarily or exclusively done in high-income countries and found increased rates of vaccine administration in non-Hispanic and non-Black participants [28-32]. Vaccination rates were also significantly higher in participants who were older and in those who had private insurance [31]. The relatively new nature of the vaccine and differing cultural attitudes toward vaccination may have played a role in these observed trends.

Overall, the RSVpreF vaccine was found to be well tolerated in pregnant women, with the most common side effects being mild to moderate local reactions. Adverse events were generally similar between vaccine and control groups; however, there was some indication that vaccine administration may be associated with HDP, which requires further investigation.

Discussion

Summary of Evidence

The RSVpreF vaccine’s efficacy has been thoroughly studied and proven to lower the risk of RSV in neonates, but data on side effects associated with the administration of the vaccine to pregnant patients are limited [16,17]. In this scoping review, we examined the potential side effects associated with the administration of the FDA-approved Pfizer RSVpreF vaccine to the pregnant patient, rather than the neonate. For this review, maternal safety was assessed via two domains: reactogenicity and adverse effects. The selected articles we reviewed provided valuable information on the observed immune reactions after administration to the pregnant patient [28-32]. The findings of this review are indicative of the growing field of interest in maternal vaccinations and the increased use and dissemination of this newly approved vaccine.

When analyzing the elements of our review question, our review was able to adequately address the maternal safety of vaccine administration by the identification of several long-term randomized control trials and a retrospective cohort study that closely followed pregnant women who had received the Pfizer RSVpreF vaccine [28-32]. Our review highlighted the general tolerability of maternal RSVpreF vaccination, with maternal side effects often being limited to local reactogenicity in the week following vaccine administration [28-30,32]. The included studies found that vaccine side effects were generally limited to injection site reactions and comparable between vaccine and control groups, with only one study reporting a significant increase in headaches and muscle pain in the vaccine group [30]. Reactions were often mild and limited to injection site pain, erythema, and tenderness [28-30,32]. These findings support the tolerance of vaccine administration in expectant patients.

In addition to assessing reactogenicity, our review also uncovered discrepancies in assessed AEs and SAEs associated with vaccine administration. In early vaccine trials, the occurrence of AEs and SAEs was found to be similar across control and vaccine groups [28-30,32]; however, upon vaccine approval, a later cohort study reported an association between vaccine use and the development of HDP [31]. These findings indicate that while the vaccine is generally well tolerated, research on maternal side effects, especially HDP, is still needed.

Our review uncovered a significant gap in data on the maternal side effects of RSVpreF vaccination. Throughout our review, while several studies were found exploring the efficacy of RSVpreF in preventing neonatal LRTI and possible associations with possible neonatal side effects, only five articles were identified that included maternal side effects and safety as outcomes of interest. Even in the few articles identified, maternal safety was often explored as a secondary focus, with neonatal vaccine efficacy being the main investigative focus [28-30,32]. While the little data reported thus far is promising in supporting the safety of maternal RSV vaccination, caution is warranted as the scope of research remains limited.

Clinical Implications of the Scoping Review

The FDA currently recommends RSV vaccination for women who are at 32-36 weeks of gestation during the local RSV season as part of routine prenatal care [5]. The findings of this review support these guidelines and the general safety of maternal RSVpreF vaccination. In establishing the current scope of data assessing the safety of maternal RSVpreF vaccination, this review may help to increase patient confidence in maternal vaccination and minimize the rates of vaccine hesitancy in expectant patients. Implications of these prospective changes could help decrease the well-established socio-economic burdens of RSV infection in infants [3] and decrease the potential development of childhood asthma and other chronic complications of RSV [11,12].

Limitations of the Scoping Review

There are major limitations in this review that warrant acknowledgment. There was a significant shortage of literature on the safety of maternal RSVpreF vaccination, with only five studies meeting inclusion criteria. This was likely due to a combination of the only recent FDA approval and limited widespread administration of the vaccine. This limited sample size may impact the generalizability of this review's findings. Additionally, these studies mostly excluded high-risk pregnancies and were often conducted in higher-income populations that typically report a lesser burden of RSV infection in newborns [28-32]. The lack of inclusion of high-risk pregnancies in these studies may have underrepresented the AEs associated with vaccination, and health providers should take this into account when counseling expectant patients with high-risk pregnancies. Furthermore, while some studies were broad and multinational [28,30], others were only conducted in a single country with limited diversity of study populations [29,31,32]. Study size was also a limitation, as these studies varied significantly in scope, with some having a very limited number of participants. Finally, there is currently no research on the long-term effects of Pfizer RSVpreF. All of this together restricts the applicability and sustainability of the results in a diverse, global setting.

Future Directions

Future research on this topic should prioritize long-term longitudinal studies in diverse patient populations and should include patients with high-risk pregnancies. Moreover, research should particularly focus on the potential association of HDP, as was noted in one study [31]. Research aimed at exploring these outcomes will hopefully provide clarity and reassurance to health providers and patients alike.

## Conclusions

This scoping review concludes that the Pfizer RSVpreF maternal vaccine is generally well tolerated in pregnant women, but further research is needed to elucidate the relationship between vaccination and HDP. Strong evidence supports the vaccine's efficacy in preventing RSV-associated LRTI in neonates; however, gaps remain in understanding its broader maternal health implications, particularly in high-risk pregnancies and underrepresented populations. More diverse research to assess potential disparities in vaccine administration and outcomes should be performed. Future research should focus on long-term maternal health effects, the vaccine’s impact on high-risk pregnancy, and potential associations of hypertensive disorders of pregnancy to ensure a comprehensive safety profile and equitable access.

## Appendices

Appendix 1

Embase Search Strategy

**Table 4:** Embase search strategy (09/14/2024): 464 results.

#1	respiratory syncytial virus vaccine'/de OR 'pf 06928316'/exp
#2	respiratory syncytial virus vaccin*':ab,ti,kw OR 'respiratory syncytial pneumovirus vaccin*':ab,ti,kw OR 'rsvpref*':ab,ti,kw OR 'rsv vaccin*':ab,ti,kw
#3	pregnancy'/exp
#4	pregnan*':ab,ti,kw OR 'maternal':ab,ti,kw
#5	#3 OR #4
#6	#1 OR #2
#7	#5 AND #6

Appendix 2

Ovid MEDLINE Search Strategy

**Table 5:** Ovid MEDLINE search strategy (09/14/2024): 316 results.

1	exp Respiratory Syncytial Virus Vaccines/
2	('respiratory syncytial virus vaccin*' or 'respiratory syncytial pneumovirus vaccin*' or 'rsvpref*' or 'rsv vaccin*').ab,ti,kf.
3	exp Pregnancy/
4	('pregnan*' or 'maternal').ab,ti,kf.
5	1 or 2
6	3 or 4
7	5 and 6

Appendix 3

Web of Science Core Collection Search Strategy

**Table 6:** Web of Science Core Collection search strategy (09/14/2024): 268 results.

1	TS=(“respiratory syncytial virus vaccin*” OR “respiratory syncytial pneumovirus vaccin*” OR “rsvpref*” OR “rsv vaccin*”)
2	TS=(“pregnan*” OR “maternal”)
3	#1 AND #2

Appendix 4

CINAHL Search Strategy

**Table 7:** CINAHL search strategy (09/14/2024): 114 results. CINAHL: Cumulative Index to Nursing and Allied Health Literature.

S1	(MH "Respiratory Syncytial Viruses")
S2	TI ( “respiratory syncytial virus vaccin*” OR “respiratory syncytial pneumovirus vaccin*” OR “rsvpref*” OR “rsv vaccin*” ) OR AB ( “respiratory syncytial virus vaccin*” OR “respiratory syncytial pneumovirus vaccin*” OR “rsvpref*” OR “rsv vaccin*” )
S3	(MH "Pregnancy+")
S4	TI ( “pregnan*” OR “maternal” ) OR AB ( “pregnan*” OR “maternal” )
S5	S1 OR S2
S6	S3 OR S4
S7	S5 AND S6

Appendix 5

 *Cochrane Central Search Strategy*

**Table 8:** Cochrane search strategy (09/14/2024): 72 results.

S1	TI ( “respiratory syncytial virus vaccin*” OR “respiratory syncytial pneumovirus vaccin*” OR “rsvpref*” OR “rsv vaccin*” ) OR AB ( “respiratory syncytial virus vaccin*” OR “respiratory syncytial pneumovirus vaccin*” OR “rsvpref*” OR “rsv vaccin*” )
S2	TI ( “pregnan*” OR “maternal” ) OR AB ( “pregnan*” OR “maternal” )
S3	S1 AND S2

Appendix 6

ClinicalTrials.gov Search Strategy

**Table 9:** ClinicalTrials.gov search strategy (09/14/2024): 25 results.

1	Other terms: “respiratory syncytial virus vaccin*” OR “respiratory syncytial pneumovirus vaccin*” OR “rsvpref*” OR “rsv vaccin*”
2	Other terms: “pregnan*” OR “maternal”
3	Other terms: (“respiratory syncytial virus vaccin*” OR “respiratory syncytial pneumovirus vaccin*” OR “rsvpref*” OR “rsv vaccin*”) AND (“pregnan*” OR “maternal”)
